# An outline of the management and prevention of postoperative ileus: A review

**DOI:** 10.1097/MD.0000000000038177

**Published:** 2024-06-14

**Authors:** Othman Iskander

**Affiliations:** a Department of Surgery, Faculty of Medicine, Jazan University, Saudi Arabia.

**Keywords:** acupuncture, colorectal surgery, Daikenchuto, laparoscopic surgery, lidocaine, nonsteroidal anti-inflammatory drugs, Postoperative ileus

## Abstract

Postoperative ileus (POI) is a prevalent surgical complication, which results in prolonged hospitalization, patient distress, and substantial economic burden. The literature aims to present a brief outline of interventions for preventing and treating POI post-surgery. Data from 2014 to 2023 were gathered from reputable sources like PubMed, PubMed Central, Google Scholar, Research Gate, and Science Direct. Inclusion criteria focused on studies exploring innovative treatments and prevention strategies for POI, using keywords such as novel POI treatments, non-pharmacological prevention, POI incidence rates, POI management, and risk factors. The findings revealed that integration of preventive measures such as coffee consumption, chewing gum, probiotics, and use of dikenchuto within enhanced recovery programs has significantly reduced both the frequency and duration of POI, without any adverse effects, with minimally invasive surgical approaches showing promise as an additional preventive strategy. While treatment options such as alvimopan, NSAIDs, and acupuncture have demonstrated efficacy, the use of lidocaine has raised concerns due to associated adverse effects. The ongoing exploration of novel therapeutic strategies such as targeting the mast cells, vagal nerve stimulation and tight junction protein, and prokinetic-mediated instigation of the cholinergic anti-inflammatory trail not only holds promise for enhanced treatment but also deepens the understanding of intricate cellular and molecular pathways underlying POI. POI presents a complex challenge in various surgical specialties, necessitating a multifaceted management approach. The integration of preventive and treatment measures within enhanced recovery programs has significantly reduced POI frequency and duration.

## 1. Introduction

Postoperative ileus (POI) emerges as a prevalent problem after abdominal surgery, contributing to extended hospitalization, patient distress, and a considerable economic burden on patients due to escalated healthcare resource utilization and declining health status.^[1]^ The frequency of POI in the initial week following surgery exhibits substantial variation, estimated to range from 3% to 50%, largely attributable to the dearth of consensus on defining abnormal recovery duration for intestinal motility.^[2]^ Central characteristics of POI involve impaired bowel function and reduced motility, which can result in ineffective movement of intestinal contents. Symptoms may include abdominal distension, decreased bowel sounds, constipation, and difficulty resuming oral intake. If these symptoms persist, complications such as dehydration, electrolyte imbalance, pneumonia, and sepsis may occur.^[3]^ Risk factors for POI include heavy blood loss, old age, and imbalanced diet. These complications not only prolong hospital stays but also elevate 30-day readmission rates and pose a risk of mortality.^[4]^ Despite the integration of enhanced recovery protocols, the prevalence of POI remains variable based on the specific surgical procedure, ranging from 7% to 27%, after colorectal surgery, demonstrating peak occurrence.^[5]^ Conversely, the prevalence of POI following laparoscopic surgery stands at 2.5%.^[6]^ Hysterectomy, as a surgical procedure, carries a POI incidence of 2%.^[7]^ Furthermore, a meta-analysis revealed that the prevalence of POI following colorectal surgery was 10.3%.^[8]^

The recuperation period from abdominal surgery, like colectomy, typically ranges from 3 to 5 days. Nevertheless, the initiation of POI prolongs hospital stays, increases patient morbidity, and raises healthcare expenses. While factors like surgical stress, inflammation, bowel functioning, and anesthetics play a role in contributing to POI, precise understanding of its pathogenesis remains limited. Clinically, POI enhances postoperative length of stay, hampers wound curing, and heightens the risk of ventral hernia development. Financially, POI is assessed to incur at least $750 million in annual healthcare expenditure in the USA.^[9]^ Similarly, recent data from New Zealand revealed a 71% surge in hospital costs for patients experiencing prolonged stay.^[10]^

The mechanism behind POI is believed to involve various factors, including surgical interventions, the use of opioids, disruptions in autonomic function and the gastrointestinal hormonal system.^[11]^ The Enhanced Recovery after Surgery proposed several preventive measures for POI, encompassing strategies such as avoiding bowel preparation, shortening preoperative fasting periods, opting for laparoscopic approaches, refraining from abdominal drains, limiting intravenous fluids, and promptly removing the nasogastric tube and bladder catheter. Despite these recommendations, clear guidelines, and policies for addressing POI have yet to be firmly established.^[12]^ Hence, this literature aims to provide the available evidence concerning the efficacy of prophylactics and treatments in the management of POI.

## 2. Methodology

The reviewed data, spanning from 2014 to 2023, was obtained from various reputable sources such as PubMed, PubMed Central, Google Scholar, Research Gate, and Science Direct. The inclusion criteria focused solely on studies that explored innovative treatments and prevention strategies for POI after surgical treatments. Consequently, studies that were unrelated to POI or investigated other surgical complications and other inaccessible studies were excluded. The search was conducted using specific keywords such as “novel treatments for POI,” “non-pharmacological preventions for POI,” “incidence rate of POI,” “management of POI,” “risk factors related to POI,” and “minimum invasive surgical techniques to reduce POI.”

## 3. Result and discussion

### 3.1. Prevention of POI

#### 3.1.1. Coffee consumption

Coffee, a widely consumed drink, has diverse effects on the body. Caffeine, a known adenosine receptor antagonist can reduce motility. Dulskas et al^[13]^ found that coffee without caffeine accelerated bowel movement compared to regular coffee, potentially resulting from the generation of novel active constituents during the decaffeination process, such as chlorogenic acids, which may contribute to the stimulation of gastric acid production and promote peristalsis, the coordinated contraction of muscles that move food through the digestive system. While the precise biological mechanisms remain unclear, it is plausible that coffee stimulates intestinal motility while inhibiting abdominal discomfort, distension, and pain – factors contributing to prevention of POI. It stimulates the release of certain hormones, such as gastrin, which can play a role in regulating gastrointestinal motility.^[14]^

Moreover, combining effective analgesia with coffee-induced gastrointestinal tract motility aids patient mobilization, reducing the likelihood of postoperative intestinal paralysis, and promoting surgical retrieval. A recent meta-analysis by Kane et al^[15]^ encompassing 6 clinical trials demonstrated that coffee intake post-surgery enhanced gastrointestinal function recovery by approximately 93%, hastening the time to first stool passage and first solid food intake (*P* < .03) and shortened the hospital stay (1.2 days vs 1.3 days, respectively), in the control group. Notably, no adverse reactions were observed with early postoperative coffee consumption.

In a randomized controlled trial by Güngordük et al, women undergoing gynecologic oncology surgery who consumed coffee 3 times every day experienced a 12-hour reduction in time to flatus (*P* < .001), a 1.3-day decrease in time to solid food tolerance (*P* < .001), a 12-hour acceleration in first bowel movement time (*P* < .001), and a 1-day shorter hospital stay (*P* = .003), compared to the control group. Multivariate analysis identified postoperative coffee consumption as an independent protective factor against the prevention of POI.^[16]^ Another meta-analysis, focusing on laparoscopic gynecologic surgery, revealed that postoperative coffee intake led to a 92% reduction in first flatus time, a 93% reduction in first defecation time, and a 93% reduction in solid diet tolerance time, contributing to a 95% prevention of POI (Table 1).^[17]^

**Table 1:** Summary of non-pharmacological prophylactics of POI.

Intervention	Author (study)	Sample size	Surgical procedure	Dose and time	Outcomes	Complications	Conclusion
Coffee consumption	Kane et al, 2020^[15]^	563	Elective colorectal surgery	3 times a day	Time to first stool passage and first solid food intake (*P* < .03)	Nil	Coffee intake post-surgery enhanced gastrointestinal function recovery by approximately 93%
	Gungorduk et al, 2017^[16]^	114	Laparotomy	3 times a day	12-h reduction in time to flatus, 1.3-d decrease in time to solid food tolerance, 12-h acceleration in the time to first bowel movement, and 1-d shorter hospital stay	Nil	Coffee intake also improved the GIT function in women undergoing gynecologic oncology surgery (*P* = .003)
	Yang et al, 2022^[17]^	312	Elective laparoscopic colonic resection	100 mL coffee thrice a day	93% reduction in the first flatus, defecation, and tolerance of solid diet duration	Nil	Reduced POI incidence rate of about 95%
Chewing gum	Jernigan et al, 2014^[20]^	109	Laparotomy	–	Nausea occurred only in 31.4% of patients due to surgical complications	Prevalence of POI was 4.6% due to surgical complication	Reduced the incidence rate of POI by about 95.4%
	Nanthiphatthanachai et al, 2020^[21]^	82	Gynecological cancer surgery	1 piece for 15 min, 3 times daily	Time to first flatus and defecation (24.7 ± 35.4 h and 3 ± 3.5 versus h)	Nil	Reduce POI (*P* = .023)
	Lambrichts et al, 2017^[23]^	40	Colorectal surgery	2 mg, after 2 h preoperatively thrice a day	Nicotine gum demonstrated a quicker onset of initial bowel movements and a faster adaptation to solid food compared to the normal gum group (3.50 d vs 4.50 d)	Nil	Nicotine gum can prevent POI to 70%
Probiotics	Bajeramajic, et al 2019^[25]^	78	Elective colectomy	Probiotic strains of *Lactobacillus rhamnosus, Bifidobacterium bifidum, Lactobacillus acidophilus, Lactobacillus plantarum, Lactobacillus casei, Bifidobacterium lactis, Streptococcus thermophilus,* and *Bifidobacterium breve* 3 d after surgery to 30 d	Reduced POI upto 12.5%	Nil	Probiotic strains prevent POI incidence by about 87.5%
	Komatsu et al, 2016^[27]^	379	Laparoscopic colorectal resection	100-mL MILMIL-S contained 1 × 10^10^ *Bifidobacterium breve* and 80-mL Yakult Ace contained 4 × 10^10^ living *Lactobacillus casei*	17.3% of patients received synbiotics acquired POI following surgery in contrast to the control group (22.7%) patients	Nil	Synbiotics also prevent POI after surgical interventions by 82.7%
	Yang et al, 2016^[26]^	60	CRC surgery	*Lactobacillus acidophilus* (≥1.0 × 10^7^ cfu/g), *Bifidobacterium longum* (≥1.0 × 10^7^ cfu/g), and *Enterococcus faecalis* (≥1.0 × 10^7^ cfu/g) 5 d before and 7 d after CRC resection operation	Improved duration to first flatus (3.63 vs 3.27 and first defecation (4.53 vs 3.87, lower incidence of diarrhea (26.67%, related to the placebo group (53.33%)	Short-term bacteremia due to *E faecalis*	Probiotics can reduce the risk of POI after colorectal surgery (*P* = .0352)
Minimal surgical interventions	Garfinkle, 2019^[29]^	9528	Loop ileostomy surgery	–	POI was observed in 16.7% of patients	Small bowel obstruction was 3%, *C difficile* diarrhea/colitis, and hospital stay time was 5 d	This surgical intervention reduced POI by about 83.3%
	Malibari et al, 2021^[30]^	120	Laparoscopic or open colectomy	–	Incidence of POI in the laparoscopic group was 7.2%, compared to 20% in the open colectomy group	Nil	Laparoscopic surgery reduced POI incidence by about 92.8%
	Li et al, 2020^[7]^	1017	Hysterectomy	–	POI observed in abdominal hysterectomy (10.6%), laparoscopic hysterectomy (7.8%), and vaginal hysterectomy (11.3%) (*P* = .279)	Anesthesia, dysmenorrhea	Overall incidence of POI was 9.2%
Daikenchuto (DKT)	Ishizuka et al, 2017	1134	Gastrointestinal cancer surgery	15 g daily for 21 d	Significant reduction in POI rates in the DKT-treated group (48%)	Nil	DKT prevented POI by about 52%
	Oyama, et.al. 2020^[34]^	904	Retroperitoneum dissection	7.5 g/d	The onset of ileus was 3.5% in DKT consuming group as compared to the control groups (4.8% and 16.4%)	Nil	The occurrence of POI decreased by 45% in the group that received DKT
	Shimada et al, 2015^[35]^	209	Hepatic resection	15 g/d, 3 times a day	First bowel movement was 88.2 h in TU-100 group	Nil	POI demonstrated a notable increase (*P* = .001), accompanied by a reduction in CRP levels

CRC = confined colorectal cancer, CRP = C-reactive protein, DKT = Daikenchuto, POI = postoperative ileus.

#### 3.1.2. Gum chewing

Chewing gum, a type of sham ingestion, triggers the cephalic-vagal reflex similar to oral food intake, stimulating motility in the duodenum, stomach, and rectum with a reduced risk of nausea and vomiting.^[18]^ Additionally, gum chewing escalates plasma concentrations of peptides like gastrin, neurotensin, and pancreatic polypeptide, as well as duodenal alkali secretion, thereby promoting gastrointestinal functions.^[19]^ A study by Jernigan et al^[20]^ did not report any incidence of POI in patients who consumed chewing gum in contrast to the control group (n = 58). Chewing gum following gynecologic surgery involving a laparotomy is considered safe and has been associated with reduced incidences of nausea and POI.

In a randomized controlled trial investigating postoperative flatus and defecation time duration, the intervention group (n = 40) received sugar-free chewing gum after the first day of surgery, chewing 1 piece for 15 minutes, 3 times daily, until achieving the milestones. The intervention group exhibited significantly shorter times to first flatus and defecation compared to the control group (24.7 ± 35.4 hours vs 52.92 ± 21.97 hours and 3 ± 3.5 hours vs 77.98 ± 34.59 hours, respectively).^[21]^ In a systematic analysis, 95% of studies reported improved recovery of bowel function, including shortened duration to first flatus and reduced length of hospital stay. Studies also proposed that nicotine gum has a dual stimulation of the vagus nerve, with one arising from physiological processes during chewing and another from the pharmacological impact of nicotine intake.^[22]^ In a randomized controlled trial, the duration of initial bowel movement and duration to solid food tolerance for a minimum of 24 hours, was observed to be shorter in the nicotine gum cohort compared to the group using regular gum (average, 3.50 days [3.00–4.25] vs 4.50 [3.00–7.25]). Nevertheless, it is imperative to conduct further investigations with more extensive participant samples to thoroughly evaluate the holistic clinical implications of using nicotine gum (Table 1).^[23]^

#### 3.1.3. Probiotics

Probiotic use in patients undergoing gastrointestinal invasion has demonstrated efficacy in reducing postoperative infectious complications. Numerous trials, particularly focused on colorectal surgery, have consistently shown a significant reduction in infectious complications with perioperative probiotic intervention. Moreover, probiotic administration has been associated with a decrease in inflammatory markers and cytokine levels.^[24]^ In a randomized controlled prospective study involving 78 patients with colorectal adenocarcinoma undergoing elective colectomy, results indicated a 12.5% reduction in POI specifically related to tumor localization. Other cases of POI were attributed to the severity of surgical wounds.^[25]^

Moreover, a study revealed that probiotics significantly improved first flatus duration (3.63 vs 3.27, *P* = .0274) and first defecation time (4.53 vs 3.87, *P* = .0268). The probiotics also exhibited a significantly lower occurrence of diarrhea (26.67%) as compared to the placebo group (53.33%; *P* = .0352). However, this improvement holds crucial clinical implications, particularly in decreasing short-term complications such as bacteremia due to *Enterococcus faecalis*.^[26]^ On the contrary, the primary outcome with synbiotics has shown the growth of infectious complications within 30 days, particularly surgical site infection. However, synbiotics treatment was found to positively impact microbial balance and organic acid levels post-surgery, with potential benefits in suppressing potentially pathogenic species. The research findings indicated that although synbiotics did not show effectiveness in decreasing infectious complications, they did exhibit favorable outcomes in terms of microbial balance and organic acid levels, leading to a successful reduction in POI following surgical interventions (Table 1).^[27]^

#### 3.1.4. Minimal invasive surgical approach

Minimally invasive surgical measures have gained widespread acceptance in recent years, significantly impacting surgical practices for both surgeons and patients. The occurrence of POI after colorectal surgery appears to be lower compared to other techniques. Laparoscopic methods, recognized for their minimal tissue manipulation, decreased surgical stress, and trauma, are widely adopted across various medical specialties due to their association with quicker patient recovery. This approach has become a norm in diverse surgical procedures. It is believed that laparoscopic techniques can alleviate the duration and intensity of ileus, resulting in short hospital stays. Unlike open surgery, several studies have indicated that minimally invasive procedures, such as laparoscopy, are correlated with reduced postoperative complications, including POI.^[28]^

A systematic review involving 9528 patients found that only 8% experienced POI after loop ileostomy surgery due to pathophysiology or patient condition.^[29]^ In a retrospective study of 120 participants undergoing either laparoscopic or open colectomy, the occurrence of POI in the laparoscopic group was 7.2%, compared to 20% in the open colectomy group. The laparoscopy group exhibited earlier tolerance of a regular diet and improved results regarding postoperative difficulties. Additionally, the laparoscopic approach was related to previous ambulation and proved more cost-effective based on hospital stay duration.^[30]^

Another surgical procedure, hysterectomy, which involves the removal of the uterus, can cause POI development by initiating a complex cascade of physiological responses, including inflammation, cytokine release, and neural signaling alterations within the gastrointestinal tract. These factors collectively result in temporary paralysis or sluggishness of bowel muscles, impeding the normal propulsive movement of intestinal contents. Moreover, the use of anesthesia, pain medications, and other perioperative factors can further contribute to the slowing down of bowel motility. Furthermore, the analysis of 1017 patients undertaking benign hysterectomy revealed a 9.2% prevalence of POI. However, this occurrence showed no substantial variation across 3 surgical methods, namely abdominal hysterectomy (10.6%), laparoscopic hysterectomy (7.8%), and vaginal hysterectomy (11.3%) (*P* = .279). Risk factors associated with POI included anesthesia, dysmenorrhea, duration of operation, and previous cancer (Table 1).^[7]^

#### 3.1.5. Daikenchuto

Daikenchuto (DKT), a traditional Japanese herbal medicine comprising dried ginger, ginseng, and zanthoxylum fruit, has been identified to improve intestinal motility.^[31]^ Animal model investigations have elucidated its favorable influence on POI, attributing this effect to its anti-inflammatory properties, particularly its interaction with nicotinic acetylcholine receptors, promoting smooth muscle contractions and improving intestinal peristalsis. Additionally, it exhibits anti-inflammatory effects, reducing the release of inflammatory markers that can contribute to POI. Overall, Daikenchuto’s multifaceted action on the enteric nervous system and inflammation makes it a potential therapeutic option for preventing POI and promoting faster recovery after surgery.^[32]^ A recent systematic analysis in Japan with 1134 participants (including 588 DKT-treated individuals), yielded a substantial decrease in POI rates in the DKT-treated group (RR = 0.58, *P* = .04, *I*^2^ = 48%)^[33]^ (Table 1).

Zingiberis Siccatum Rhizoma has been identified as a constituent of DKT that activates the alpha-7 Nicotinic Acetylcholine Receptor (7AChR) by stimulating transient receptor potential ankyrin 1 (TRPA1) channel in the enterochromaffin cells. This activation initiates the stimulation of the 5-hydroxytryptamine 4 receptor (5-HT4) in the enteric nervous system, thereby regulating gastrointestinal motility, facilitating peristalsis, and improving bowel movements.^[33]^ In a study of 904 patients undergoing gynecological surgery, DKT significantly reduced ileus onset, particularly in patients receiving both an adhesion-inhibitory barrier and oral DKT. This preventive effect contributed to a 45% decrease in severe ileus requiring surgery, enhancing patients’ postoperative quality of life.^[34]^ Another study showed that TU-100 (DKT) intake, following hepatic resection significantly reduced serum CRP levels (*P* = .001) and accelerated first bowel movement duration by approximately 88.2 hours.^[35]^

### 3.2. Management of POI

#### 3.2.1. Alvimopan

Alvimopan acts as a mu-opioid receptor antagonist, selectively blocks opioid effects on the gastrointestinal tract and helps to enhance the retrieval of normal bowel function, reducing POI occurrence. It is typically administered perioperatively and is particularly beneficial in patients at risk of prolonged ileus, contributing to earlier resumption of oral intake and overall faster recovery after surgery.^[36]^ A specific study on radical cystectomy for bladder cancer where alvimopan was provided perioperatively, provided a significant reduction in the likelihood of ileus-related readmissions (*P* = .041), and hospital costs by −$2709 (*P* = .003), suggesting alvimopan as a potentially cost-effective strategy.^[37]^

Another double-blind, randomized trial in participants after cytoreductive surgery and hyperthermic intraperitoneal chemotherapy (CRS/HIPEC) revealed a 10% decrease in POI incidence compared to 37% in the placebo group.^[38]^ However, a retrospective analysis of laparoscopic abdominal surgeries revealed no statistically substantial change in the occurrence of POI between the cohorts administered alvimopan and those not given the treatment (2.7% vs 4.3%, *P* = 1). These findings were plausibly attributed to the comparable severity of patients and the administration of a lower drug dosage (Table 2).^[39]^

**Table 2:** Management of POI after surgical intervention.

Treatment	Author	Sample size	Surgical treatment	Dose and time	Outcomes	Complications	Results
Alvimopan	Huang et al, 2020^[37]^	1087	RC	10 d	Reduced POI (*P* = .041) and hospital costs by −$2709 (*P* = .003	Nil	Alvimopan is considered a potentially cost-effective strategy after RC to reduce POI incidence rate
	Baumgartner et al, 2021^[38]^	62	Cytoreductive surgery and hyperthermic intraperitoneal chemotherapy	12 mg twice a day is recommended for a duration of 7 d following surgery	First flatus time (79 h vs 64 h), bowel movement (89 h vs 67 h), solid food tolerance (149 h vs 117 h), and discharge (233 h vs 241 h)	Nil	POI incidence decreased about 10% as compared to 37% in the placebo group
	Kokan et al, 2021^[39]^	84	Laparoscopic abdominal surgery	–	Duration of hospitalization (5.4 d compared to 5.4 d, *P* = .49), postoperative stay duration (5 d vs 4.9 d, *P* = .44), time until initiation of oral diet (0.9 d vs 0.4 d, *P* = .16), and time to bowel movement (1.8 d vs 2.2 d, *P* = .32)	POI found 2.7% in the Alvimopan group as compared to the control group 4.3%	Results revealed that no statistically significant difference was found in the incidence of POI between groups treated with alvimopan and those without (*P* = 1)
Acupuncture	Ruan et al, 2019^[41]^	80	Myomectomy, hysterectomy, ovarian cystectomy, surgery for ectopic pregnancy and adnexectomy	Massage of acupoints included Zusanli, Neiguan, Zhongyu, and Sanyinjiao, thrice daily	The first bowel movement in the acupuncture group was 11.01 ± 3.25 h vs 18.01 ± 4.22 h in the control, first anal exhaust (control group, 28.05 ± 5.80 versus observation group, 17.65 ± 3.25 h) and first defecation (control group, 45.96 ± 7.80 h; observation group, 34.01 ± 7.59 h)	Nil	Results indicated that POI was treated at 78.75% compared to the control group and elevated levels of motilin and cholecystokinin were also noted for their role in stimulating gastrointestinal motility
	Yang et al, 2022^[43]^	129	Elective colorectal resection	(EA) at ST36 or ST25 at 2/100 Hz lasted for 30 min, 4 d	EA at ST36 shortened the first flatus and defecation time (*P* = .02) and (*P* = .26) at ST25	Nil	POI was reduced to 93.3% after EA
	Wang et al, 2023^[44]^	248	Laparoscopic bowel resection	*Zhongwan* (RN12) and bilateral acupoints *Tianshu* (ST25), *Zusanli* (ST36), and *Shangjuxu* (ST37) at 2 per 100 Hz	Time to first defecation was 76.4 h in the EA group and 90.0 h in the Sham Acupuncture (SA) group, time to first flatus was 44.3 in EA vs 58.9 h in SA, solid food intake in EA was 181.8 h vs 190.3 in SA	Nil	POI in the EA group was approximately 90% less compared to the sham EA group
Lidocaine	Dai et al, 2020^[46]^	115	Gastrointestinal tumor surgery	1.5 mg/kg lidocaine for 90 d after surgery	Time to flatus, bowel movement, hospitalization days, and associated expenses in the lidocaine-receiving group compared to the control group (*P* < .05)	Drowsiness and dry mouth	IV lidocaine infusion can reduce acute postoperative pain, promote postoperative gastrointestinal function recovery, and improve postoperative comfort without severe adverse effects
	Foo et al, 2020^[48]^	4525	GIT surgery	1.5 mg/kg/h for no longer than 24 h	Lidocaine reduced POI after surgery (*P* = .01)	6.8% of participants faced somnolence, metallic taste, dizziness, agitation, nausea, peri-oral numbness, tinnitus, and tremor	Results revealed that lidocaine causes adverse effects in patients after surgery
	Herzog et al, 2020^[49]^	60	Robot-assisted colorectal surgery	1.5 mg/kg/h of Lidocaine infusion continued until 2 h after end of surgery	There is no substantial disparity in postoperative morphine use at both 24 and 72 h for relieving pain associated with POI (*P* > .05)	POI occurred in 17 patients and required re-operation	The findings underscore the nuanced considerations surrounding the implementation of intravenous lidocaine in the context of POI management
NSAIDs	STARSurg Collaborative, 2022	5240	Elective gastrointestinal surgery	3 d after surgery	No significant difference was found in gastrointestinal recovery between NSAID recipients and non-recipients (4.6 d vs 4.8 d; *P* = .360)	Anastomotic leak (*P* = .349) or acute kidney injury (*P* = .666)	NSAIDs did not expedite gastrointestinal recovery after colorectal surgery, however, their use was deemed safe
	Brolet et al, 2021^[51]^	8611	Colorectal surgery	–	NSAIDs were found to be significantly reduced postoperative complications (21.1% vs 29.2%), and a shorter hospital stay (5.0 d instead of 6.0)	POI was found in 2.1% of patients, kidney infection was found in 29.2% of patients	NSAIDs had a positive impact in reducing POI (*P* < .001)
	Milne et al, 2018^[52]^	515	Elective colorectal surgery	–	Flatus time (*P* = .02), stool pass time (*P* < .001) and diet tolerance time (*P* < .001)	Diclofenac increased the leak rate by 54%	NSAIDs helped accelerate the healing process of GIT after planned colorectal surgery

EA = electroacupuncture, NSAIDs = nonsteroidal anti-inflammatory drugs, POI = postoperative ileus, RC = radical cystectomy.

#### 3.2.2. Acupuncture

Electroacupuncture (EA) has emerged as a noninvasive technique recently with the potential to alleviate pathological intestinal inflammation through vagal nerve stimulation. Vagal nerve stimulation is being explored as a promising therapeutic strategy for managing inflammatory responses. The electrical stimulation of cervical vagal efferent has shown the capability to attenuate systemic inflammation by activating the α7 nicotinic acetylcholine receptor (α7nAChR) found on tissue-resident macrophages and other immune cells. The distinct anti-inflammatory capabilities help alleviate the pathological intestinal inflammation in POI and help manage POI symptoms including vomiting or nausea.^[40]^

In another study, massage of the acupoints following gynecological surgery significantly reduced durations for defecation, food tolerance and flatus, along with POI (*P* < .01). Although the mechanisms behind acupuncture on gut function remain unclear, Ruan et al^[41]^ introduced a hypothesis proposing that acupoint massage could potentially stimulate the autonomic nervous system through the vagus nerve. This stimulation, in turn, is suggested to regulate gastrointestinal motility by modulating several gastrointestinal hormones, resulting in a 78.75% decrease in POI compared to the control group. Additionally, the study noted elevated levels of motilin and cholecystokinin, recognized for their role in stimulating gastrointestinal motility.

Animal studies have also suggested that abdominal acupoints may delay bowel motility, while lower limb acupoints may promote it. Notably, Zusanli (ST36) acupoints on the lower limbs and Tianshu (ST25) acupoints on the abdomen are commonly used for gastrointestinal disease treatment.^[42]^ In an investigation focusing on individuals experiencing laparoscopic elective colorectal resection in colorectal cancer, the application of EA at the acupoint ST36 demonstrated notably shorter durations for the initiation of the first flatus and defecation in comparison to those receiving standard care exclusively. However, no statistically significant difference in these outcomes was observed when EA was applied at the ST25 acupoint, though 93.3% of patients were discharged without POI complications, and no serious adverse events were reported. It is worth noting that patients in the ST36 group were also taking i-Mo-Tang, a Chinese patent medicine for digestive motility enhancement, which might have influenced the outcomes.^[43]^ Using the same acupoints, another investigation concentrating on the efficacy of EA versus sham EA demonstrated a low prevalence of POI in EA group, approximately 90% less compared to the sham EA group. Importantly, no severe adverse events were reported in either group (Table 2).^[44]^ The choice of acupoints in EA was suggested as a contributing factor to these positive outcomes.

#### 3.2.3. Lidocaine

To enhance postoperative recovery, intravenous infusion of lidocaine has emerged as a valuable intervention targeting the initial phase of POI. The mechanism of action by which lidocaine exerts its beneficial effects in alleviating POI is multifaceted. The mechanism of action of lidocaine involves the inhibition of voltage-gated sodium channels and blocking the initiation and conduction of nerve impulses. This local anesthetic effect is thought to suppress the hyperexcitability of sensory nerves in the gut, ultimately reducing pain and inflammation associated with surgical trauma. These anti-inflammatory properties potentially modulate the release of inflammatory mediators like interleukin-6 (IL-6) and tumor necrosis factor-alpha involved in POI pathogenesis. Furthermore, lidocaine has been implicated in attenuating sympathetic nervous system activity, which may help restore the balance between sympathetic and parasympathetic inputs to the gut, facilitating a more coordinated and efficient postoperative recovery of gastrointestinal function. This dual action on both sensory and autonomic pathways makes lidocaine a promising therapeutic option for managing POI.^[45]^

This approach has shown benefits within the Enhanced Recovery After Surgery procedure by mitigating the activity of the sympathetic nervous system. In addition, perioperative intravenous lidocaine infusions indicated a marked decrease in various parameters, including the return of flatus, bowel movement, hospitalization days, and associated expenses in the lidocaine-receiving patients in contrast to the control group (*P* < .05). Importantly, this progress was achieved with minimal adverse effects, primarily limited to dizziness.^[46]^ In the latest Cochrane review, data from 50 out of 68 studies reported lidocaine-related adversities, where 23 studies showed the absence of significant events, while the remaining 27 studies documented minor side effects, including but not limited to lightheadedness, peri-oral numbness, tinnitus, drowsiness, and bradycardia.^[47]^

Also, Fu et al^[48]^ revealed that somnolence, metallic taste, dizziness, agitation, nausea, peri-oral numbness, tinnitus, and tremor were observed in 6.8% of participants after lidocaine consumption. In another prospective, double-blinded trial undergoing robot-assisted colorectal surgery, intravenous lidocaine treatment did not yield the desired results in postoperative cumulated morphine intake at 24 or 72 hours for alleviating POI-related pain (*P* > .05). However, within the lidocaine group, some cases required reoperation for POI. These cases included 2 instances of anastomotic leakages, perineal wound infection, and small bowel perforation through abscess. These findings underscore nuanced considerations surrounding the implementation of intravenous lidocaine in the context of postoperative pain management and POI mitigation (Table 2).^[49]^

#### 3.2.4. Nonsteroidal anti-inflammatory drugs

Nonsteroidal anti-inflammatory drugs (NSAIDs) can reduce POI through the inhibition of the cyclooxygenase (COX) enzyme, responsible to produce prostaglandins associated with inflammation and pain. By inhibiting COX-2, NSAIDs suppress the synthesis of prostaglandins and help mitigate the excessive inflammatory response triggered by surgical trauma, reducing the associated intestinal smooth muscle dysfunction and inflammation. Consequently, the modulation of prostaglandin levels by NSAIDs contributes to improved gastrointestinal motility and function, ultimately aiding in reducing and preventing POI. However, the use of NSAIDs is limited by their analgesic shortcomings and notable side effects, such as platelet dysfunction, GI bleeding, and renal issues.^[28]^

A prospective investigation conducted by an international collaborative group led by students and trainees showed no significant difference in gastrointestinal recovery between NSAID recipients and non-recipients (4.6 days vs 4.8 days; *P* = .360). No notable variations were found in the anastomotic leak rate (5.4% vs 4.6%; *P* = .349) and acute kidney complication (14.3% vs 13.8%; *P* = .666) between the groups. Notably, NSAIDs did not expedite gastrointestinal recovery after colorectal surgery, however, their use was deemed safe and correlated with a reduced need for postoperative opioids.^[50]^

Another retrospective study revealed a reduction in postoperative complications, including 2.1% POI and 29.2% kidney infection, suggesting that perioperative NSAID use contributes to improved adherence, fewer in-hospital complications, and a short length of stay (5 days vs 6 days) undergoing colorectal surgery.^[51]^ A recent meta-analysis highlighted an association of diclofenac with a 54% increased leak rate, while ketorolac demonstrated no such correlation. This difference can be attributed to different absorption, distribution, metabolism, and excretion patterns of both NSAIDs in the body. The evidence on this matter is inconclusive and needs further investigation (Table 2).^[52]^

#### 3.2.5. Potential future pharmacological approaches for cellular and molecular targets

##### 3.2.5.1. Mast cells

The crucial juncture in the inflammatory cascade of the ileus revolves around the activation and degranulation of mast cells. This process can be effectively impeded by targeting nerve growth factor (NGF). Mast cells undergo activation when NGF binds the tropomyosin receptor kinase A (trkA), a high-affinity NGF receptor leading to inhibited mast cell degranulation and reduced expression of IL-6.^[53]^ This process diminishes gastrointestinal inflammation and contributes to the prevention of POI. Similarly, Jardí et al^[54]^ discovered that the NGF receptor antagonist, K252a, alleviated mast cell hyperactivity induced by oral ovalbumin, consequently mitigating the effects of colonic contractile alterations.

Furthermore, investigations into Capsaicin and Calcitonin Gene-Related Peptide antagonists have illuminated the association between mast cells and afferent neurons through the involvement of receptor activity-modifying protein 1 (RAMP1). Nonetheless, the precise mechanism remains incompletely comprehended. In murine models, the Capsaicin and Calcitonin Gene-Related Peptide receptor antagonist olcegepant (BIBN4096BS) demonstrated a reduction in IL-1β and IL-6 mRNA expression of muscularis externa by inhibiting mast cell degranulation, following 3 hours of surgery.^[55]^ A pilot clinical trial demonstrated that the administration of 12 mg of ketotifen significantly reduced gastric emptying time. This effect was achieved through the reduction of mast cell degranulation and the inhibition of histamine and various cytokines. Consequently, it affected smooth muscle activity in the stomach, leading to 18% gastric retention and improved coordination of the digestive process. In comparison, the placebo group receiving 4 mg of ketotifen showed gastric retention of 3%, highlighting the dose-dependent impact on gastric emptying.^[56]^

##### 3.2.5.2. Vagal pathway

The vagal nerve reduces POI by the secretion of acetylcholine (ACh), inhibiting resident macrophages through the attachment of α7nAChR and modulating the immune response within the gastrointestinal environment exhibiting reduced inflammatory reaction in cytokine production, recruitment of inflammatory markers and intestinal muscle, consequently expediting transit recovery. A study on the depressed murine POI model revealed that the activation of the vagus nerve effectively suppresses macrophage activation, mitigates inflammation in smooth muscles of the intestines, and proves to be an effective intervention for addressing POI. Additionally, the research indicates that Vagus Nerve Stimulation emerges as a potential therapeutic tool with anti-inflammatory, antinociceptive, and antidepressive properties, likely due to its positive impact on the brain-gut connection. While the vagus nerve indirectly interfaces with immune cells in the intestinal wall, anatomical investigations have emphasized proximity of cholinergic neuronal fibers to macrophages within the gastrointestinal tract, occurring in both the myenteric plexus and lamina propria. Prokinetics like prucalopride have exhibited promise in animals and humans for monitoring inflammation and treating POI by stimulating the vagus nerve.^[57]^

##### 3.2.5.3. Maintaining the integrity of tight junction proteins

Tight junction proteins (TJPs) play a crucial role in regulating gut paracellular permeability, a key factor in POI recovery. It forms a barrier between adjacent cells in the intestinal lining, playing a crucial role in regulating paracellular permeability and maintaining the intestinal barrier integrity to prevent POI. Surgical stress and inflammation can disrupt tight junctions, leading to increased permeability and potential complications such as POI. In a POI study model, the administration of glutamine demonstrated a modulatory effect on claudin-1 and claudin-2 (TJPs), crucial for maintaining intestinal tissue permeability. The precise mechanisms by which glutamine interacts with TJPs and inhibits POI are not yet fully understood. However, it is hypothesized that glutamine may play a role in preserving the integrity of tight junctions, thereby preventing the interruption of the intestinal wall and inhibiting gastrointestinal tract inflammation. Moreover, glutamine demonstrated anti-inflammatory effects by modulating paths such as NF-κB, a signal transducer for inflammatory markers like IL-6. These mechanisms may collectively contribute to the prevention or reduction of POI. The ability of glutamine to influence these pathways suggests its potential as a therapeutic agent in mitigating inflammation in the intestinal tract.^[58]^

##### 3.2.5.4. Prokinetic mediated activation mechanism

Prokinetic agents are small peptide drugs that enhance gastrointestinal motility and can play a crucial role in mitigating POI. By promoting smooth muscle contractions in the digestive tract, these agents accelerate the passage of contents through the intestines, thereby preventing stasis and reducing the likelihood of ileus. Furthermore, prokinetic-mediated initiation of the cholinergic anti-inflammatory pathway offers an additional therapeutic dimension. Cholinergic anti-inflammatory pathway, driven by the vagus nerve and acetylcholine release, helps modulate inflammatory responses. By harnessing this pathway, a dual benefit is achieved – improved gastrointestinal motility and dampened inflammatory reactions. This integrated approach potentially leads to quicker recovery and enhanced postoperative outcomes.

Mosapride functions by targeting the 5-hydroxytryptamine 4 receptor within the myenteric plexus nerve. This action leads to the stimulation of ACh release at the distal extremity of vagal efferent, subsequently hindering macrophages through their α7 nicotinic ACh receptors. The released ACh, in turn, hampers the secretion of pro-inflammatory mediators like tumor necrosis factor-alpha, IL-6, MIP-2, and MIP-1α, thereby alleviating inflammation. An alternative strategy involves addressing intracellular signaling pathways within the muscularis macrophages (MMs) to mitigate the activation of transcription factors, the initiation of pro-inflammatory gene expression, and the release of chemokines and cytokines. Semapimod, an inhibitor of p38 mitogen-activated protein kinase, has shown effectiveness in reducing POI by inhibiting the expression of proinflammatory genes like macrophage inflammatory protein (MIP-1α), Interleukin-6 (IL-6), and Intercellular Adhesion Molecule (ICAM-1). This action slows down the recruitment of leukocytes, suggesting a potential therapeutic approach to counteract the impact of POI.

## 4. Conclusion

POI poses a complex challenge across various surgical specialties, prompting a multifaceted approach to its management. The integration of preventive measures such as coffee consumption, chewing gum, probiotics and DKT within enhanced recovery programs has significantly reduced both the frequency and duration of POI, without any adverse effects. While treatment options such as alvimopan, NSAIDs and acupuncture have demonstrated efficacy, the use of lidocaine raises concerns due to associated adverse effects. The ongoing exploration of novel therapeutic strategies such as targeting the mast cells, vagal nerve stimulation and tight junction protein and prokinetic-mediated stimulation of the cholinergic anti-inflammatory mechanism not only holds promise for enhanced treatment but also deepens the understanding of the intricate cellular and molecular mechanisms underlying POI.

## 5. Future aspects

Long-term effects and potential complications of some preventive and therapeutic interventions, especially those that are relatively novel or less studied, require more extensive investigation. Addressing these limitations will not only enhance our understanding of POI but also contribute to the progress of more effective and personalized strategies for its prevention and management.

## Author contributions

**Conceptualization:** Othman Iskander.

**Data curation:** Othman Iskander.

**Formal analysis:** Othman Iskander.

**Funding acquisition:** Othman Iskander.

**Investigation:** Othman Iskander.

**Methodology:** Othman Iskander.

**Project administration:** Othman Iskander.

**Resources:** Othman Iskander.

**Software:** Othman Iskander.

**Supervision:** Othman Iskander.

**Validation:** Othman Iskander.

**Visualization:** Othman Iskander.

**Writing** – **original draft:** Othman Iskander.

**Writing** – **review & editing:** Othman Iskander.
